# Long-term ileoanal pouch survival after pouch urinary tract fistulae

**DOI:** 10.1007/s10151-024-02948-w

**Published:** 2024-06-25

**Authors:** T. Uchino, E. P. Lincango, O. Lavryk, J. Lipman, H. Wood, K. Angermeier, S. R. Steele, T. L. Hull, S. D. Holubar

**Affiliations:** 1 Department of Colorectal Surgery, Digestive Diseases Institute, Cleveland Clinic Main Campus, 9500 Euclid Ave, A30, Cleveland, OH 44122 USA; 2 Department of Urology, Glickman Urology and Kidney Institute, Cleveland Clinic, Cleveland, OH USA

**Keywords:** Ileoanal pouch, Ulcerative colitis, Crohn’s disease, Ileal pouch-anal anastomosis, Urinary fistula

## Abstract

**Background:**

Ileoanal pouch is a demanding procedure with many potential technical complications including bladder or ureteral injury, while inflammation or stricture of the anastomosis or anal transition zone may lead to the formation of strictures and fistulae, including to the adjacent urethra. Pouch urinary tract fistulae are rare. We aimed to describe the presentation, diagnostic workup, and management of patients with pouch urinary at our center.

**Methods:**

Our prospectively maintained pouch registry was queried using diagnostic codes and natural language processing free-text searches to identify ileoanal pouch patients diagnosed with any pouch-urinary tract fistula from 1997 to 2022. Descriptive statistics and pouch survival using Kaplan–Meier curves are presented. Numbers represent frequency (proportion) or median (range).

**Results:**

Over 25 years, urinary fistulae were observed 27 pouch patients; of these, 16 of the index pouches were performed at our institution [rate 0.3% (16/5236)]. Overall median age was 42 (27–62) years, and 92.3% of the patients were male. Fistula locations included pouch-urethra in 13 patients (48.1%), pouch-bladder in 12 patients (44.4%), and anal-urethra in 2 (7.4%). The median time from pouch to fistula was 7.0 (0.3–38) years. Pouch excision and end ileostomy were performed in 12 patients (bladder fistula, *n* = 3; urethral fistula, *n* = 9), while redo ileal pouch-anal anastomosis (IPAA) was performed in 5 patients (bladder fistula, *n* = 3; urethral fistula, *n* = 2). The 5-year overall pouch survival after fistula to the bladder was 58.3% vs. 33.3% with urethral fistulae (*p* = 0.25).

**Conclusion:**

Pouch-urinary tract fistulae are a rare, morbid, and difficult to treat complication of ileoanal pouch that requires a multidisciplinary, often staged, surgical approach. In the long term, pouches with bladder fistulae were more likely to be salvaged than pouches with urethral fistulae.

## Background

Restorative proctocolectomy with ileal pouch-anal anastomosis (IPAA) is considered the preferred option for the management of ulcerative colitis (UC), familial adenomatous polyposis (FAP), and other conditions which require total proctocolectomy, and allows the majority of patients to avoid a permanent ileostomy [1]. However, IPAA is a technically demanding procedure and may be associated with significant complications. The incidence of complications associated with IPAA has been reported to be as high as 50% [2]. If severe, these complications may result in pouch failure requiring permanent diversion or pouch excision in up to 15% of cases [3–7].

Pelvis sepsis is a dreaded postoperative, technical complication as the development of pouch-related fistulae to various locations including the perineum and gynecologic structures, and suboptimal long-term pouch function may result [8]. The presence or later development of Crohn’s disease (CD)-related complications, including inflammation or stricture of the anastomosis, rectal cuff, anal canal, and/or anal transition zone (ATZ) also play a role in the development of many pouch-related fistulas [9]. Additionally, there have been reports indicating that a long-term determinant of pouch failure is pouch fistulae, as a result of chronic leaks [10]. The most common pouch fistulae are perianal and pouch (ano)-vaginal, while and enterocutaneous fistulas are less common. Pouch urinary tract fistulae (PUTF) represent a small minority (< 10%) of all postoperative pouch fistulae, and a paucity of data has been reported in the literature with which to guide treatment [6, 8, 11]. Therefore, we aimed to describe patient characteristics, preoperative workup, treatment, outcomes, and long-term pouch survival for PUTF experienced at our institution.

## Methods

This study is a single-center, descriptive case series of patients diagnosed with PUTF. Our institutional review board (IRB)-approved ileal pouch registry, which included pouches constructed between 1983 and 2022 and also those referred to our center for pouch revision, was used to identify PUTF using the following codes to search the electronic medical record: ICD-9 codes 599.1, 593.82, 596.2; ICD-10 code N32.2 or 36.0. Potential patients were identified from the electronic health record using natural language processing including operative reports. Pouch registry data was then supplemented by manual abstraction of additional variables including symptoms, diagnostic imaging findings, fistula source, treatment, and outcomes. We excluded patients with small bowel-to-urinary tract fistula due to trauma and patients without IPAA. Our primary endpoint was pouch survival after fistula surgery; pouch failure was defined as permanent re-diversion (including diverting ileostomies that were never closed) without pouch excision or pouch excision with permanent ileostomy. Inflammation of the ATZ/rectal cuff was evidenced by erythema, ulceration, and friability as noted by the surgeon at time of exam under anesthesia. The diagnosis of CD was made by the treating surgeon and gastroenterologist with documented, pathologic inflammation of the rectal cuff and/or pouch.

**Fig. 1:**
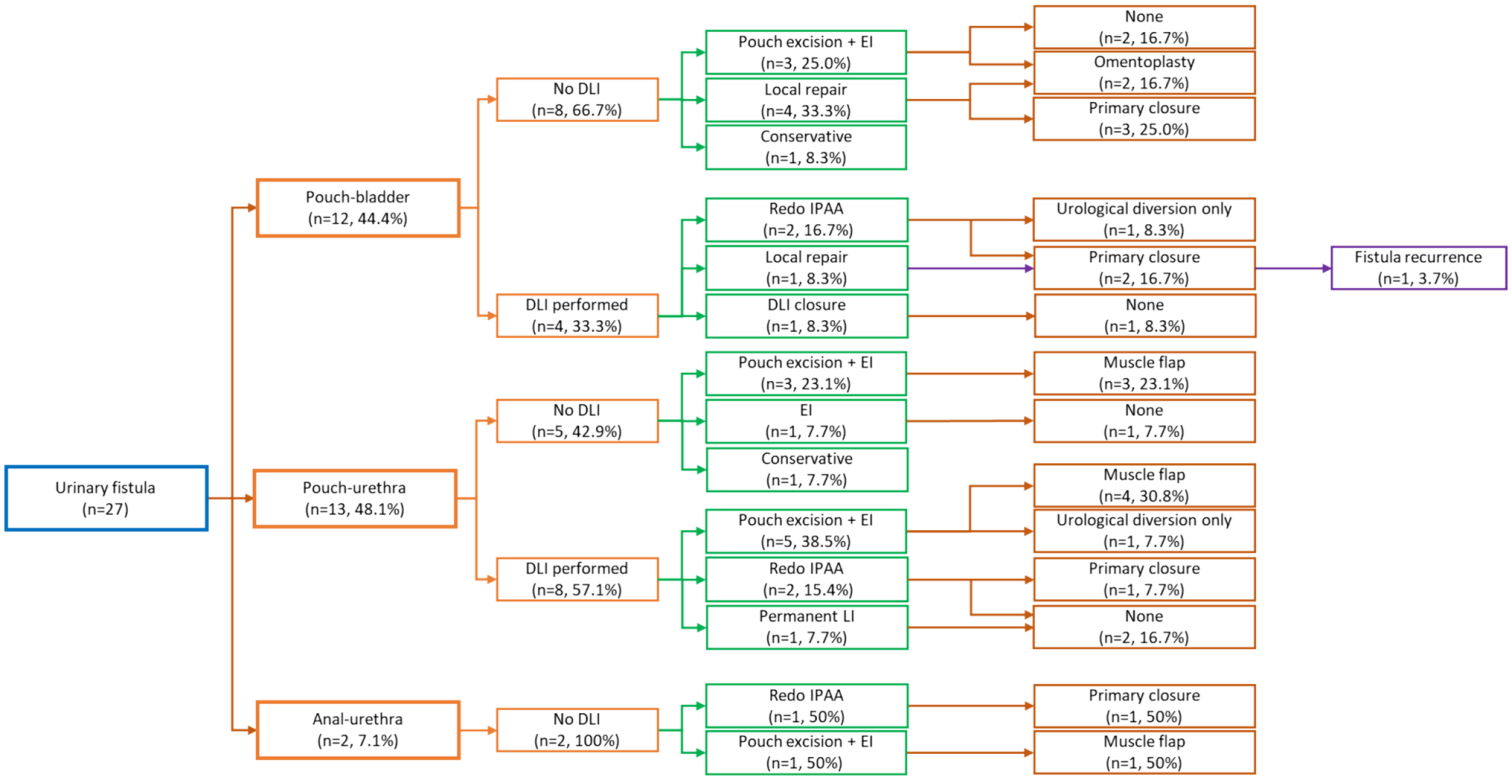
Flowchart of management in patients with urinary fistula. *EI* end ileostomy, *LI* loop ileostomy, *DLI* diverting loop ileostomy, *IPAA* ileal pouch anal anastomosis

A flowchart of management in patients with urinary fistula in shown in Fig. 1.

### Statistical analysis

Categorical variables were reported as frequencies and percentages. Continuous variables were reported as median with range. The Mann–Whitney *U* test was used to compare continuous variables and the factors between bladder fistulae and other fistulae. The overall pouch survival, considering competing risk factors such as gender and fistulae site, was calculated using the method of Kaplan and Meier, with number of patients at risk reported, and it was compared by log-rank test. EZR version 1.61 software (2022, Tochigi-ken, Japan) were used to perform all analyses [12].

## Results

### Patient characteristics

A total of 27 patients with PUTF were identified and included. Of these, 16 of the index IPAA were performed at our institution, representing 16/5236 (0.3%) of our pouch cohort. The median age at index IPAA was 29 years old (11–53), median age at PUTF surgery was 41 years old (39–49), 25 (92.6%) were male. Indication for IPAA included UC (*n* = 23, 85.2%), FAP (*n* = 3, 11.1%), and CD in the remaining case (*n* = 1, 3.7%). Among the 27 patients who underwent an index IPAA, 20 (74.1%) had a 2-stage IPAA, 5 (18.5%) had a 3-stage IPAA, and 2 (7.4%) had a 1-stage IPAA. Two cases experienced postoperative technical issues after index IPAA: a perforation at the site of diverting loop ileostomy closure and an anastomotic stricture within 2 months. The median time from index IPAA surgery to fistula presentation was 7.0 years (Table 1). Bladder fistulae had a shorter time from index IPAA to fistula presentation than urethral fistulae (2.5 vs. 14.0 years, *p* = 0.01, Fig. 1). A total of 25 operations were performed by 17 colorectal and 10 urologic surgeons over a 30-year period; two conservative cases who were managed non-operatively with adalimumab and observation.

**Table 1:** Patient characteristics

Variables	*N* = 27 (100%)
Age at index IPAA, years	29 (11–53)
Age at PUTF surgery, years	41 (39–49)
Gender, male	25 (92.6%)
BMI, kg/m^2^	23.3 (17.2–31)
Indication for index IPAA	
Ulcerative colitis	23 (85.2%)
Familial adenomatous polyposis	3 (11.1%)
Crohn’s disease	1 (3.7%)
Index pouch construction at Cleveland Clinic	16 (59.3%)
IPAA stage	
1	2 (7.4%)
2	20 (74.1%)
3	5 (18.5%)
Anastomosis technique at index IPAA	*N* = 15
Handsewn	7 (46.7%)
Stapled	8 (53.3%)
Time from index IPAA to fistula, years	7 (0.3–38)
Presenting symptoms	
Pneumaturia	17 (63.0%)
Fecaluria	7 (25.9%)
Abdominal pain	7 (25.9%)
Nausea/vomiting	2 (7.4%)
Pyuria	2 (7.4%)
Hematuria	1 (3.7%)
Asymptomatic	1 (3.7%)
Number of symptoms	
1	16 (59.3%)
≥ 2	11 (40.7%)
Fistula site^a^	
Pouch-urethral	14 (51.9%)
Pouch-bladder	11 (40.7%)
Ano-urethral	2 (7.4%)

Figures represent frequency (proportion), median (range)

^a^Both female patients had bladder fistulae

### Presenting symptoms

Symptoms present at the time of diagnosis of the PUTF are summarized in Table 1. The most common presenting symptom was pneumaturia, present in 17 (63.0%) patients, while 7 (25.9%) reported fecaluria, and 7 (25.9%) had abdominal pain; 1 (3.7%) patient was asymptomatic at the time of diagnosis of the fistula which was found incidentally at the time of a follow-up pouchoscopy of the index IPAA. Sixteen patients (59.3%) had one symptom and 11 (40.7%) had two or more symptoms.

### Diagnostic modalities

The diagnostic modalities are summarized in Table 2. Preoperative examination included cystoscopy in 24 (88.9%) patients, pouchoscopy in 19 (70.4%), CT scan in 16 (59.3%), cystogram and urethrogram in 11 (40.7%), and pelvic MRI in 9 (33.3%) patients. The rate of patients with positive examination findings was highest for MRI (100% [*n* = 9/9], 62.5% [*n* = 15/24] for cystoscopy, 57.9% [*n* = 11/19] for pouchoscopy, 54.5% [*n* = 6/11] for cystogram and urethrogram, and 68.8% [*n* = 11/16] for CT scan).

**Table 2:** Diagnostic modalities used to identify origin of the PUTF

Method of diagnosis of fistula site	Positive findings *N* (%)
MRI	9/9 (100%)
CT scan	11/16 (68.8%)
Cystoscopy	15/24 (62.5%)
Pouchoscopy	11/19 (57.9%)
Cystogram/urethrogram	6/11 (54.5%)

All patients had more than one diagnostic test

*CT* computed tomography, *MRI* magnetic resonance imaging

### Management

Fistula management is summarized in Table 3. In bladder fistula group, managements included pouch local repair in 5 (41.7%), pouch excision with end ileostomy in 3 (25%), redo IPAA in 2 (16.7%) patients, and conservative treatment in 1 (8.3%). Only 1 patient (8.3%) had preoperative urinary diversion along with their bowel diversions via a suprapubic tube (SPT). Three patients who underwent pouch excision with end ileostomy received a final diagnosis of CD.

**Table 3:** Patient characteristics at time of fistula surgery

Variables	
Bladder fistulae	*N* = 12 (44.4%)
Re-diversion before definitive surgery	4 (33.3%)
Preoperative urinary diversion, SPT	1 (8.3%)
Crohn’s disease	6 (50%)
Presence of peri-anastomotic/ATZ inflammation or stricture	1 (6.7%)
Definitive surgery for fistula	
Local repair	5 (41.7%)
Pouch excision and EI	3 (25%)
Redo IPAA	2 (16.7%)
DLI	1 (8.3%)
Conservative	1 (8.3%)
Management for urinary fistulous opening	
Primary suture	5 (41.7%)
Omental flap	2 (16.7%)
Urological diversion (SPT)	1 (8.3%)
Prolonged temporary bladder catheterization	5 (41.7%)
Urethral fistulae	*N* = 15 (55.6%)
Re-diversion before definitive surgery	8 (53.3%)
Preoperative urinary diversion, SPT	5 (33.3%)
Crohn’s disease	9 (60%)
Presence of peri-anastomotic/ATZ inflammation or stricture	8 (53.3%)
Definitive surgery for fistula	
Pouch excision and EI	9 (60%)
Redo IPAA	3 (20%)
DLI	1 (6.7%)
EI	1 (6.7%)
Conservative	1 (6.7%)
Management for urinary fistulous opening	
Gracilis interposition flap	8 (53.3%)
Primary suture	2 (13.3%)
Urological diversion only	2 (13.3%)
None	3 (20%)

Figures represent frequency (proportion), median (range)

*SPT* suprapubic tube, *ATZ* anal transitional zone, *EI* end ileostomy, *DLI* diverting ileostomy

In the urethral fistula group, management included pouch excision with end ileostomy in 9 (60%), redo IPAA in 3 (20%) patients, and conservative treatment in 1 (6.7%). Five patients (33.3%) had preoperative urinary diversion (SPT). Among the nine patients who underwent pouch excision with end ileostomy, six had inflammation of the anal canal/ATZ, and two had anastomotic strictures. The patient who did not have ATZ inflammation or anastomotic stricture had a CD-related fistula as the underlying cause. Among the four patients who underwent a redo pouch procedure, one who had ATZ inflammation did not experience fistula recurrence but ultimately required pouch excision with end ileostomy.

**Fig. 2:**
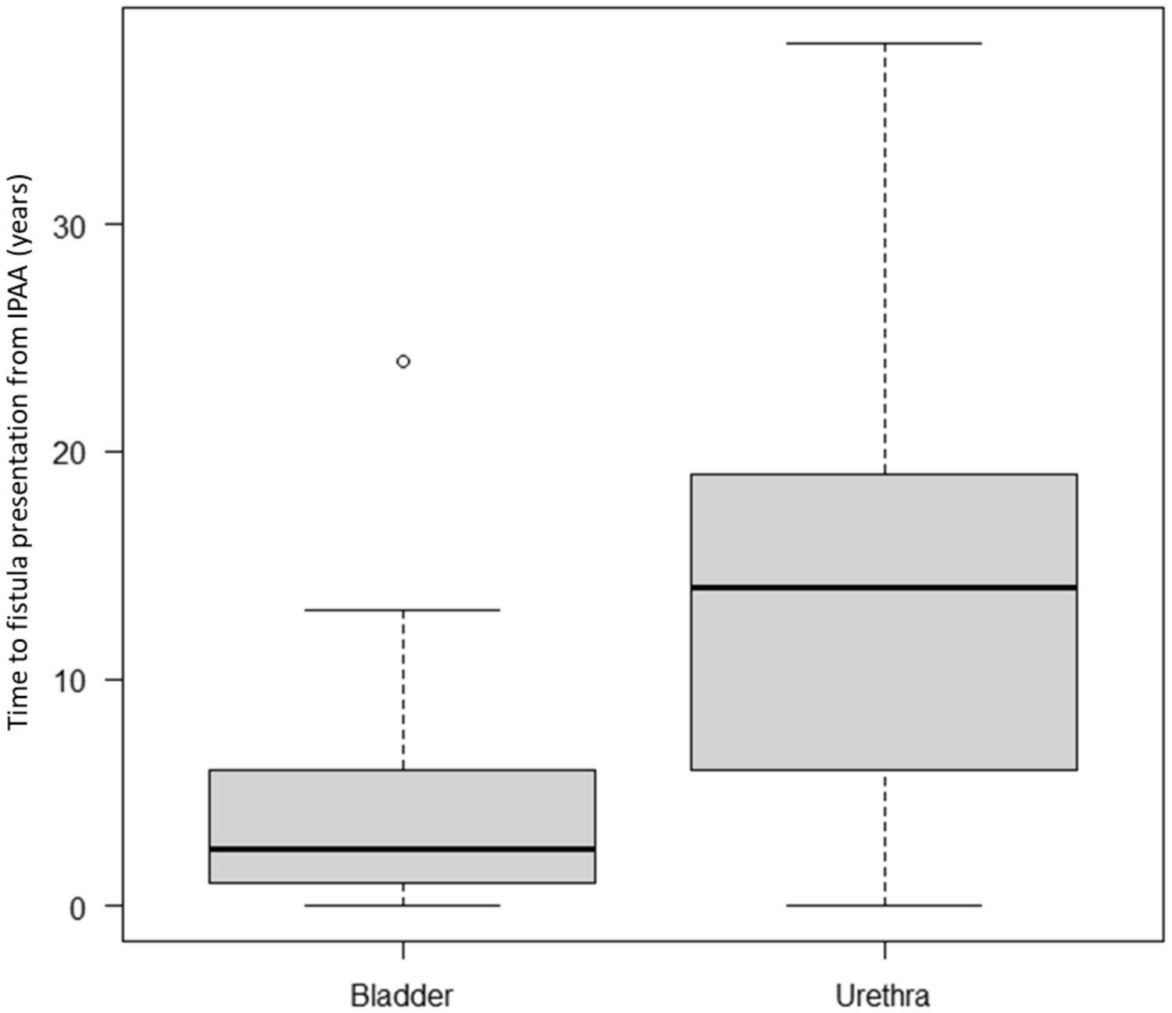
Boxplot of time to fistula presentation in patients with bladder vs. non-bladder fistulae. Dark black lines represent median (2.5 years vs. 14 years, *p* = 0.01), shaded area represents interquartile range, tails represent range, and dot represents outliers, for bladder and urethral fistulae, respectively

The relationship between definitive surgery and fistula location is summarized in Fig. 2. Overall, 12 (44.4%) patients were re-diverted before attempted definitive surgery. Temporary diverting ileostomy was more commonly performed in pouch urethral fistula (*n* = 8/15, 53.3%) patients compared to bladder fistula (*n* = 4/12, 33.3%). In patients who underwent initial re-diversion with a temporary diverting loop ileostomy, redo IPAA was performed in 4/12 (33.3%) patients. In the bladder fistula group, one patient with mild symptoms underwent conservative treatment with adalimumab for CD-like inflammation (it was found that the perineal fistula had become complex, but no evidence of urinary fistula was found). In the urethral fistula group, one patient underwent conservative treatment with adalimumab and resolved.

### Long-term outcomes

**Fig. 3:**
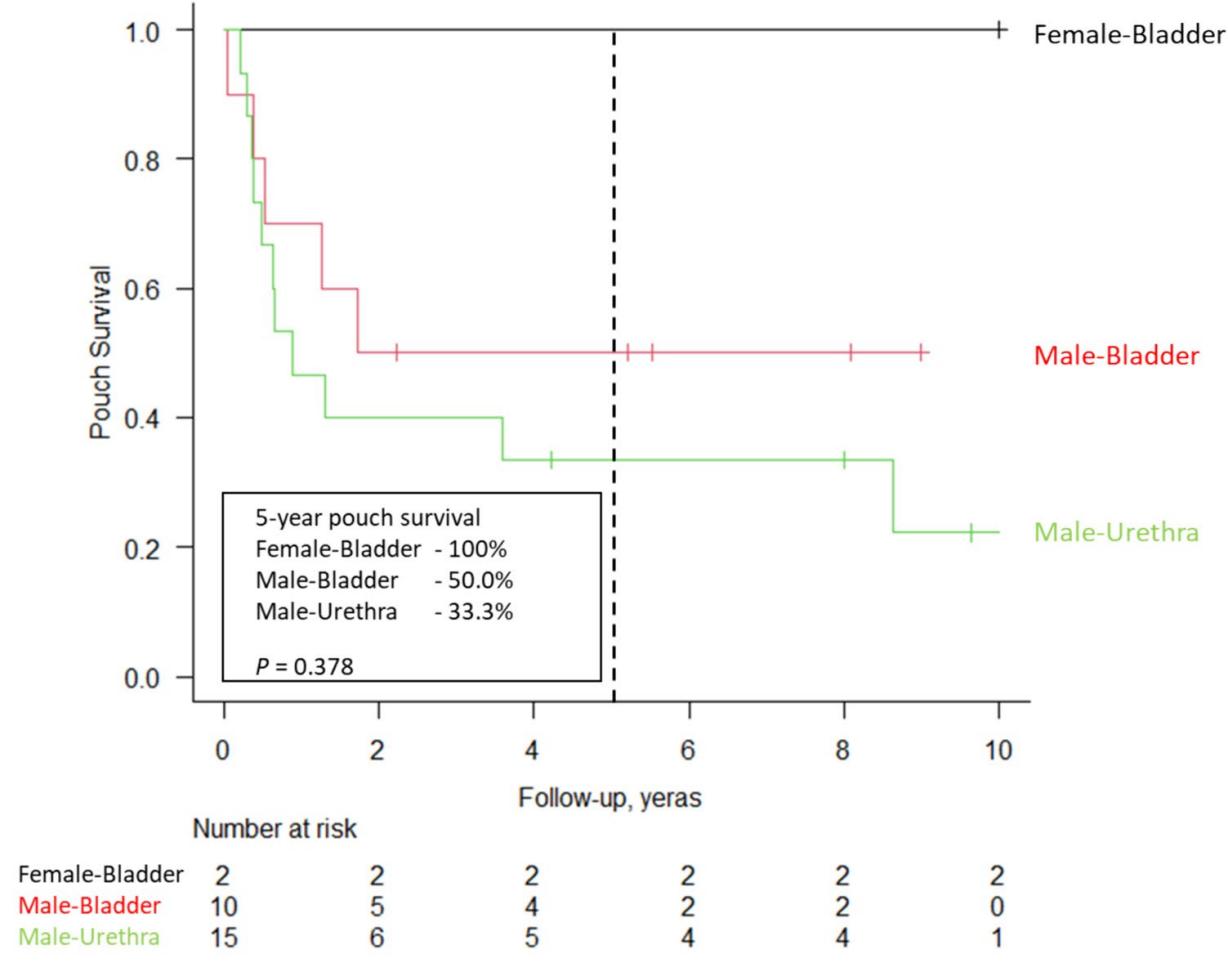
Overall pouch-survival after diagnosis of PUTF by fistula location

The overall 5-year pouch survival after PUTF presentation was 44.1%. The overall 5-year pouch survival rates after PUTF presentation with gender and fistula location from bladder fistulae and urethral fistulae were male bladder fistulae 50%, male urethra fistulae 33.3%, both female bladder fistulae cases achieved pouch survival, respectively (*p* = 0.38) (Fig. 3). After temporary diverting ileostomy surgery or definitive surgery, two patients never underwent diverting ileostomy closure with pouch left in situ, and one patient after diverting ileostomy closure followed by re-diversion with another permanent diverting loop ileostomy. There was only one case of fistula recurrence, which was managed with pouch repair for a bladder fistula. All patients with urethral fistula had resolution of their fistulae without any recurrences. All three patients who have achieved long-term pouch survival with urethral fistulae did not have ongoing issues with ATZ inflammation or stricture. Among the six patients who have achieved long-term pouch survival with bladder fistulae, only one received a final diagnosis of CD.

## Discussion

Proctocolectomy with IPAA is the procedure of choice for patients with medically refractory UC and other conditions requiring total proctocolectomy [13]. IPAA is associated with complications such as pouchitis, anal stenosis, pelvic sepsis, and fistulae in a significant number of patients. These complications are treated either with medical or surgical therapy to achieve good long-term pouch survival. Fistulae often require surgical treatment and may be recurrent. In our study, the overall 5-year pouch survival in patients with urinary fistula was 44.1% (58.3% for bladder fistulae and 33.3% for urethral), which was lower than the rates reported in previous studies. While the overall failure rate for pouches is up to 15%, the reported failure rate for complications related to fistula varies depending on the study [3–7]. Gaertner et al. found that the pouch failure in the setting of fistulae rate was 38% [14], while Tekkis et al. reported a failure rate of 21% for women and 27% for men [9]. Our study focuses only on fistulae that occur in the urinary tract after pouch surgery and excludes pouch vaginal and other fistulae; although the excluded fistulae may be more common, their inclusion may have clouded some study results making them less directly comparable. Nonetheless, these findings are significant in predicting the prognosis of pouches in patients who have undergone restorative proctocolectomy with IPAA.

Pouch-related fistulae occur in 3.4–10% of cases, with the most being perianal and pouch (ano-)vaginal, and pouch-bladder and enteric fistulas being less common [6, 11, 15]. Most patients with peri-pouch fistulas result in pouch resection or permanent ostomy [13]. The rate of redo IPAA for PUTFs at our institution was 1% (6/603) of all redo IPAA performed in the same period. Surgeons dealing with this complication need to recognize the risk factors, timing of occurrence, and location of the fistula. In a retrospective study of PUTF patients, Kjaer et al. showed that patients with more rapid fistula development were at higher risk for pouch excision (*p* = 0.007) [16]. In this study, there were only two cases of early fistula development within 6 months, and both patients underwent pouch excision. Steele et al. showed that women with sigmoid diverticulitis are less likely to develop a fistula to the bladder because of interposition of the uterus between the bladder and the colon; therefore, women with an intact uterus are also less likely to develop a pouch-bladder after IPAA surgery. Similarly, in men the urethra is in close proximity to the anal canal, while in women the vagina is interposed. Thus, in this study, only 2 (7.4%) women were observed to develop PUTF—both of which were bladder [17]. Thus male gender appears to be a strong if not the strongest risk factor for PUTF.

In terms of etiology of the fistulae, as shown in Fig. 1, the bladder fistula occurred significantly sooner after index IPAA than urethral fistula, suggesting that the bladder fistulae were more likely related to a technical complication, such as a leak from the tip of the J, while the urethral fistula may have been more likely to be related, overall, to perianal CD. While it remains unclear via retrospective chart review whether early postoperative complications after index IPAA resulted from occult tip of J leak, or if CD was a direct cause of urinary tract fistula, the fact that over half of the patients developing urinary tract fistulae had CD suggests a high likelihood that chronic inflammation may be a contributing factor to fistula formation.

Regarding diagnostic imaging, Solem et al. reported that cystoscopy and abdominal/pelvic CT were the most useful diagnostic modalities (74% and 52%, respectively) for fistulas to the urinary system in CD. In the present study, cystoscopy/CT had similar results; MRI had the most findings although only 9 patients (33.3%) had MRIs. The authors also reported pneumaturia (68%) as the most common symptom, similar to the present study (65.4%) [18].

Multiple studies have reported surgical treatments for pouch-related fistula including redo IPAA, pouch revision, fistulotomy, gracilis transposition, transanal pouch advancement flap, and conservative therapy. High success rates of gracilis muscle transposition for rectovaginal and pouch-vaginal fistulas have been reported [19]. Authors have noted that an increasing number of prior local repair attempts is associated with increased failure rates at subsequent operations. The presence of an anal canal stricture is also associated with suboptimal success rates for local fistula repair attempts [20]. A recent systematic review reported a lower recurrence rate for redo IPAA (42.1%) than for transvaginal repair or transanal ileal pouch advancement flaps (52.3% and 56.9%) [21]. Regarding gracilis muscle transposition for rectal fistulae, Korsun et al. and Zmora et al. reported one and three cases, respectively, and both groups reported that it is a potentially good treatment [22, 23].

Regarding medical management, we note that two patients were managed conservatively, without surgery, with tumor necrosis factor inhibitor (TNFi) biologic medications, one of whom (a urethral fistula) healed. We certainly advocate biologic therapy, typically with TNFi if possible, for any patient in whom a diagnosis of CD is entertained as the etiology.

The time to occurrence was significantly different between bladder and urethral fistulae in this study (bladder, 2 years vs. urethral, 14 years; *p* = 0.005). There was also a difference in the rate of diversion before definitive surgery (pouch-bladder, 36.4% vs. pouch-urethra, 72.8%; *p* = 0.18), although not significant, and a difference in the 5-year overall pouch survival after PUTF (bladder, 63.6% vs. non-bladder, 26.7%; *p* = 0.16); consequently we need to distinguish between a bladder fistula and a urethral fistula.

This study has several limitations. Firstly, this is a single-center retrospective study that is limited by relatively small numbers which precluded a formal risk factor analysis, although male gender and presence of peri-anastomotic/ATZ inflammation or stricture emerged as putative risk factors. Selection bias may also have influenced our results with surgeons selecting patients for pouch salvage vs. pouch excision on the basis of patient preference, surgeon experience, technical feasibility, and other factors. Considering that many patients with inflammation in the ATZ or anastomotic strictures underwent pouch excision, it is believed that these factors are crucial elements in the determination of the surgical approach as redo pouch procedures for CD are at risk of these issues returning and may be more definitely managed with pouch excision. Secondly, about half of the patients underwent proctocolectomy with IPAA at other institutions; therefore, the details of the surgery and the details of the onset of PUTF are incomplete, and their association was not evaluated. As a quaternary high-volume IBD referral center, our findings may not be generalizable to other centers; specifically, 40% of pouches in this series were referred from other centers, so the outcomes of pouch salvage surgery may depend on referral patterns, patient comorbidities, and center- and surgeon-related variations in practice at the time of index IPAA. Despite these limitations, our study adds incrementally to the understanding of the prognosis for patients with these difficult to treat fistulae.

For the surgeon, treating PUTF is a technical challenge, but for the patient, the impact on quality of life is significant. An important preventive step is to have experience or to build a team around an expert and well-experienced surgeon, but PUTF is quite rare and is most often treated in specialized facilities. Therefore, it is important to obtain data that can be analyzed in the literature, although there are few reports, to determine the best treatment.

## Conclusion

PUTF is a rare and severe complication that typically required major surgery for pouch salvage or symptomatic palliation. Male gender and anal canal inflammation or strictures appear to be the primary risk factors. Overall pouch survival was low at 42% because of the complexity of repair. These results are important for predicting pouch prognosis at the PUTF.

## Data Availability

The data that support the findings of this study are available from the corresponding author, SDH, upon reasonable request.
